# A new OIT protocol for severe peanut allergy

**DOI:** 10.1186/2045-7022-3-S3-P6

**Published:** 2013-07-25

**Authors:** AK Kukkonen, A Pelkonen, M Mäkelä, H Voutilainen, S Mäkinen-Kiljunen

**Affiliations:** 1 Skin and Allergy Hospital, Pediatric Department, Helsinki University Central Hospital, Helsinki, Finland

## Background

Oral immunotherapy (OIT) may reduce the risk for severe allergic reaction at accidental ingestion of peanut. We aimed at developing a peanut OIT protocol with home-based dose escalations for 6-18 year-old children with moderate to severe allergic reaction in a double-blind placebo-controlled challenge (DBPCFC).

## Methods

Our pirmary outcome was the increase in the amount of ingested peanut protein tolerated in DBPCFC by peanut OIT. Secondarily we investigated safety of OIT. Since September 2011, peanut-sensitized patients with moderate to severe allergic reaction (1) in DBPCFC were included. Bronchial hyperreactivity, exhaled nitric oxide, and food allergy related quality of life (2, 3) were evaluated pre and post OIT. Roasted and defatted peanut flour, allergenity of which was assessed using microarray inhibition, was mixed with margarine (no milk or soy) in 3 concentrations at hospital kitchen. A teaspoonful of peanut-margarine mixture weight 1.7 g. From week 20 and on, we used real peanuts. The first dose of 0.1 mg peanut protein was taken at hospital and the same dose continued daily at home. Dose escalations occurred at home every 1 to 2 weeks. The patient returned to the hospital 7 times for dose escalation. Antihistamine was taken daily until 2 weeks from reaching the maintenance dose of 4 peanuts. As safety procedures, exercise was forbidden one hour post each dose and the day of escalation, and the patients carried adrenalin auto-injectors.

## Results

In November 2012, 18 patients mean age 9 (6-16) y have entered the OIT. At DBPCFC, the median dose of peanut protein causing moderate to severe allergic reaction was 55 mg (1/4 peanut), range 5 to 255 mg, 10/18 had received 1 to 3 doses of adrenalin as treatment for anaphylactic reaction at challenge. The median (range) serum peanut specific-IgE was 89 (1.8-817) kU/L and peanut skin prick test was 10 (6-16). Fourteen children had asthma. Two patients discontinued the study; one was non-compliant and one had continuous nausea. One patient has received adrenalin after dose escalation at home; 2 patients have taken antihistamines once due to urticaria. Mild oro-pharyngeal itching requiring no medication was common.

## Conclusion

Based on our preliminary results it seems possible to develop a peanut OIT protocol with home-based dose escalations also in anaphylactic patients.

## Disclosure of interest

None declared.
